# On the Topological Phase around Conical Intersections with Tamm–Dancoff Linear-Response Time-Dependent Density Functional Theory

**DOI:** 10.1021/acs.jpca.4c02503

**Published:** 2024-06-26

**Authors:** Jack T. Taylor, David J. Tozer, Basile F. E. Curchod

**Affiliations:** † Department of Chemistry, Durham University, South Road, Durham DH1 3LE, United Kingdom; ‡ Centre for Computational Chemistry, School of Chemistry, University of Bristol, Cantock’s Close, Bristol BS8 1TS, United Kingdom

## Abstract

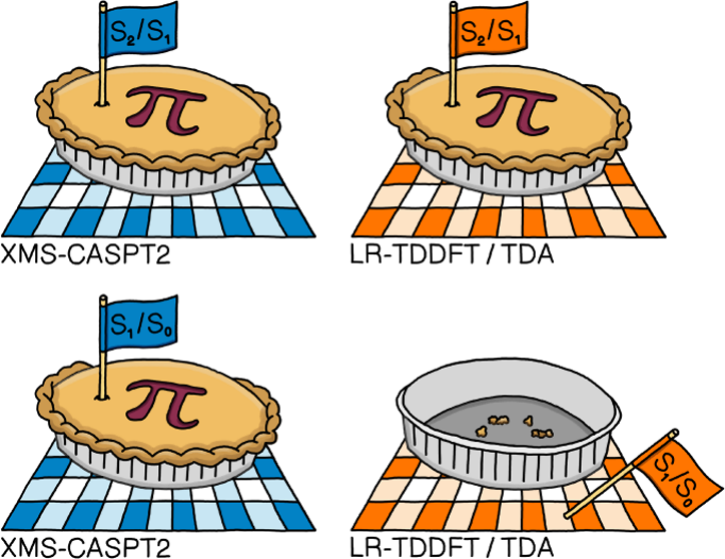

Regions of nuclear-configuration space away from the Franck–Condon geometry can prove problematic for some electronic structure methods, given the propensity of such regions to possess conical intersections, i.e., (highly connected) points of degeneracy between potential energy surfaces. With the likelihood (perhaps even inevitability) for nonadiabatic dynamics simulations to explore molecular geometries in close proximity to conical intersections, it is vital that the performance of electronic structure methods is routinely examined in this context. In a recent paper [TaylorJ. T.J. Chem. Phys.2023, 159, 214115.38059547 10.1063/5.0176140], the ability of linear-response time-dependent density functional theory within the adiabatic approximation (AA LR-TDDFT) to provide a proper description of conical intersections, in terms of their topology and topography, was investigated, with particular attention paid to conical intersections between two excited electronic states. For the same prototypical molecules, protonated formaldimine and pyrazine, we herein consider whether AA LR-TDDFT can correctly reproduce the topological phase accumulated by the adiabatic electronic wave function upon traversing a closed path around an excited-to-excited state conical intersection despite not using the appropriate quadratic-response nonadiabatic coupling vectors. Equally, we probe the ability of the ground-to-excited state intersection ring exhibited by AA LR-TDDFT in protonated formaldimine to give rise to a similar topological phase in spite of its incorrect dimensionality.

## 1 Introduction

Linear-response time-dependent density functional theory (LR-TDDFT)^1^ offers a good compromise between computational affordability and chemical accuracy. Rooted in an exact formalism^2−4^ based on the time-dependent electronic density (a much simpler alternative to the time-dependent many-electron wave function), LR-TDDFT is often considered the electronic structure workhorse for tackling the excited electronic states of medium- to large-sized molecular systems. However, in practical applications, the accuracy of LR-TDDFT is limited^5,6^ by the adiabatic approximation (AA), which results in the loss of the frequency-dependence of the exchange-correlation kernel and by the ground-state approximation to the exchange-correlation functional, for which a “zoo” of approximations exists.^7^ Even within the Franck–Condon region, AA LR-TDDFT calculations using approximate exchange-correlation functionals can therefore be unreliable, e.g., for transitions dominated by double-excitation character^8,9^ or those involving charge transfer.^10−13^ Of key importance to nonadiabatic dynamics, the description of conical intersections (CXs), points of degeneracy between two (or more) adiabatic electronic states, is another well-established case where AA LR-TDDFT can breakdown.^14−21^ Within AA LR-TDDFT, the ground (reference) state is treated on a different footing to that of the singly excited (response) states; the former is acquired variationally upon solving the Kohn–Sham equations of ground-state DFT, whereas the latter are acquired together from the Casida equation, which naturally includes coupling between them.^22^ As a result, CXs between the ground and lowest excited electronic states exhibit an incorrect topology (and/or topography) in AA LR-TDDFT, as was discussed recently in ref (21). Specifically, it was shown that for protonated formaldimine, depending on the extent of molecular distortions along the S_1_/S_0_ branching plane, AA LR-TDDFT within the Tamm–Dancoff approximation (TDA)^23^ gives either a (near-to-linear) seam of intersection^14^ or two slightly interpenetrating cones,^15^ both of which emerge from an S_1_/S_0_ intersection ring. The study in ref (21). also confirmed explicitly that for protonated formaldimine and pyrazine, AA LR-TDDFT is able to reasonably describe CXs between two excited electronic states despite using approximate linear-response (rather than the appropriate quadratic-response) derivative coupling vectors to plot the S_2_/S_1_ minimum-energy CX (MECX) branching spaces.

The analysis in ref (21). centered primarily on visualizing the potential energy surfaces (PESs) in the branching space of a given MECX [or minimum-energy crossing point (MECP)^21^] to establish its dimensionality, as well as calculating numerical parameters^24,25^ to characterize its topography (where appropriate). Electronic energies computed at “optimized” CX geometries determined using standard electronic structure codes are, however, never exactly degenerate due to finite numerical accuracy.^26^ Therefore, it is not strictly possible to establish whether a CX (rather than a narrowly avoided crossing) has been located from inspection of the PESs in the branching space alone.^27^ The existence of a CX can, however, be verified by considering the topological phase effect:^28−31^ a (real-valued) adiabatic electronic wave function accumulates an additional topological phase of π, i.e., it changes sign, as it traverses a path enclosing a CX. Thus, examining the sign of the electronic wave function obtained from a given electronic structure method along a closed path within the branching plane can be used to determine whether a CX has indeed been located. In the absence of a CX, no additional topological phase/sign change of the adiabatic electronic wave function is observed along the closed path.

The present study, however, is focused on the performance of AA LR-TDDFT/TDA, which has no formal access to the interacting electronic wave function. It is therefore necessary to examine an alternative, albeit intrinsically related, signature of CXs. Specifically, the circulation of the (first-order) nonadiabatic coupling vector, **d**_*ij*_(**R**), that is, its (vector) line integral along a closed path *C*_*n*_,
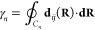
1should, in the case of an infinitesimal path, return the accumulated topological phase, i.e., it should return a value of π if the path *C*_*n*_ encloses a CX or a value of zero if the path does not enclose a CX.^26,27,32−37^ In eq 1, *i* and *j* denote the electronic states, **R** denotes the collective variable for all nuclear coordinates, and *n* labels the closed path of interest. In practical calculations, the path *C*_*n*_ will be small but not infinitesimal, so values close to, but not exactly equal to, π or zero will be obtained.

Given that **d**_*ij*_(**R**) vectors are well-defined^38^ quantities in TDDFT, they can be used within eq 1 to provide incontrovertible evidence of the presence of a CX. However, when evaluated between two excited electronic states, the **d**_*ij*_(**R**) vectors can only be defined exactly within a quadratic-response (QR) formalism.^38−43^ An interesting question therefore arises: does eq 1 return a value close to π when evaluated using AA LR-TDDFT/TDA along paths enclosing the optimized S_2_/S_1_ MECXs in protonated formalidimine and pyrazine in ref (21). despite the use of (approximate) linear-response **d**_*ij*_(**R**) vectors? We note that earlier works in connection with the pseudo wave function approximation to AA LR-TDDFT(/TDA),^44−47^ as well as AA QR-TDDFT(/TDA),^47^ have discussed the value of eq 1 in this context, all reported a value of γ_*n*_ close to π.

A second interesting question is what value will eq 1 return when evaluated using AA LR-TDDFT/TDA along a path enclosing the S_1_/S_0_ intersection ring observed for protonated formaldimine in ref (21)? In this case, exact **d**_*ij*_(**R**) vectors involving the ground electronic state can be obtained from linear-response TDDFT; however, it is the incorrect behavior of the PESs within the vicinity of the supposed degeneracy point that results in the infamous failure of LR-TDDFT in this context. As stressed most recently by Williams et al.^48^ in relation to “defective” excited-to-excited state MECXs in EOM-CCSD, the topological phase effect is only observed (i.e., eq 1 returns π) if the path *C*_*n*_ encloses an odd number of CXs; if *C*_*n*_ encloses an even number of CXs, then the topological phase effect is not observed (i.e., eq 1 returns zero). This is a more general statement than that presented above and is an important detail relevant to our present work; the AA LR-TDDFT/TDA S_1_/S_0_ intersection ring arguably comprises an infinite number of degeneracy points, not just a single point of degeneracy, so it is not immediately obvious as to what value eq 1 should take.

The purpose of this article is to address these two questions. We organize our work as follows: We begin by providing computational details, in particular highlighting how eq 1 was numerically evaluated in practice. We then compare values of γ_*n*_ from eq 1, determined using AA LR-TDDFT/TDA, with those from XMS-CASPT2 (extended multistate complete active space second-order perturbation theory), our reference electronic structure method of choice, evaluated along closed paths on the respective MECX (or MECP) branching planes for protonated formaldimine (S_2_/S_1_ and S_1_/S_0_) and pyrazine (S_2_/S_1_).

## 2 Computational Details

### 2.1 Electronic Structure

Following ref (21), the XMS-CASPT2 energies, energy gradients,^49^ and nonadiabatic and derivative coupling vectors^50^ were obtained with the BAGEL 1.2.0 program package,^51^ applying the single-state, single-reference (SS-SR) contraction scheme,^49,52^ and a real vertical shift of 0.3 au to reduce problems with intruder states. All XMS-CASPT2 calculations used the Dunning cc-pVTZ basis set.^53^ Frozen core and density fitting approximations were also employed, with the latter making use of the cc-pVTZ-jkfit auxiliary basis set from the BAGEL library. For protonated formaldimine, a (6/4) active space, including the two pairs of C–N σσ* and ππ* orbitals, was employed with a three-state averaging. For pyrazine, a (10/8) active space, comprising the six π orbitals and two nitrogen lone pairs, was used with a three-state averaging. The DFT^54−56^ and AA LR-TDDFT energies, energy gradients, and nonadiabatic and derivative coupling vectors^44,57^ were obtained using the PBE0 (global hybrid) exchange-correlation functional^58−60^ and Dunning cc-pVDZ basis set^53^ within a developmental version of the graphics processing unit (GPU)-accelerated TeraChem 1.9 program package.^61−67^ All AA LR-TDDFT calculations made use of the TDA. For brevity, we will hereafter exclude the “AA” when discussing our LR-TDDFT/TDA results. Quantities involving the ground and excited states will be labeled by (LR-TD)DFT/TDA/PBE0, whereas those involving excited states only will be labeled by LR-TDDFT/TDA/PBE0.

### 2.2 Plotting the CX Branching Space

For each S_*j*_/S_*i*_ MECX, the geometry was first optimized with XMS-CASPT2 using the gradient-projection algorithm of Bearpark et al.,^68^ on top of which the corresponding raw branching space vectors, **g**_*ij*_(**R**) and **h**_*ij*_(**R**), were computed. The raw branching space vectors were orthogonalized according to the Yarkony procedure^26,69^ and normalized [see the Supporting Information (SI) of ref (21). for branching space vector definitions] and then used to construct a two-dimensional (2D) grid of 29 × 29 geometries along the branching plane with its origin at the optimized XMS-CASPT2 S_*j*_/S_*i*_ MECX geometry. This was accomplished by appropriately scaling the nuclear distortions along the orthonormalized **x̅**_*ij*_(**R**) and **y̅**_*ij*_(**R**) vector directions and adding them in 14 increments in the positive and negative directions, respectively, spanning ±0.001 Å in both branching space directions. See Section 3.2 for details of the extended branching plane dimensions. A single-point XMS-CASPT2 energy calculation was performed at each grid-point geometry, allowing the S_*j*_ – S_*i*_ energy difference to be calculated in the vicinity of the optimized XMS-CASPT2 S_*j*_/S_*i*_ MECX geometry.

The same procedure was repeated to obtain the corresponding S_*j*_ – S_*i*_ energy difference in the region surrounding the S_*j*_/S_*i*_ MECX (or MECP) of (LR-TD)DFT/TDA/PBE0, except for two details. First, a combination of different geometry optimization algorithms was used to locate the S_*j*_/S_*i*_ MECX (or MECP) geometry in (LR-TD)DFT/TDA/PBE0, ensuring that the lowest possible electronic energy was found for these critical points, see the SI of ref (21). for details. Second, to directly compare the branching spaces obtained by XMS-CASPT2 and (LR-TD)DFT/TDA/PBE0 (i.e., two different electronic structure methods), we rotated the orthonormalized branching space vectors of (LR-TD)DFT/TDA/PBE0 within their respective branching plane to ensure maximal overlap with the reference orthonormalized branching space vectors of XMS-CASPT2. As in ref (21), these new rotated (orthonormalized) branching space vectors are denoted as **x̅**_*ij*_^′^(**R**) and **y̅**_*ij*_^′^(**R**). The rotation procedure (for the (LR-TD)DFT/TDA/PBE0 branching space vectors) and process used to orthonormalize the raw branching space vectors (of both electronic structure methods) is outlined explicitly in the SI of ref (21).

### 2.3 Calculating the Circulation of the Nonadiabatic Coupling Vector

**Figure 1:**
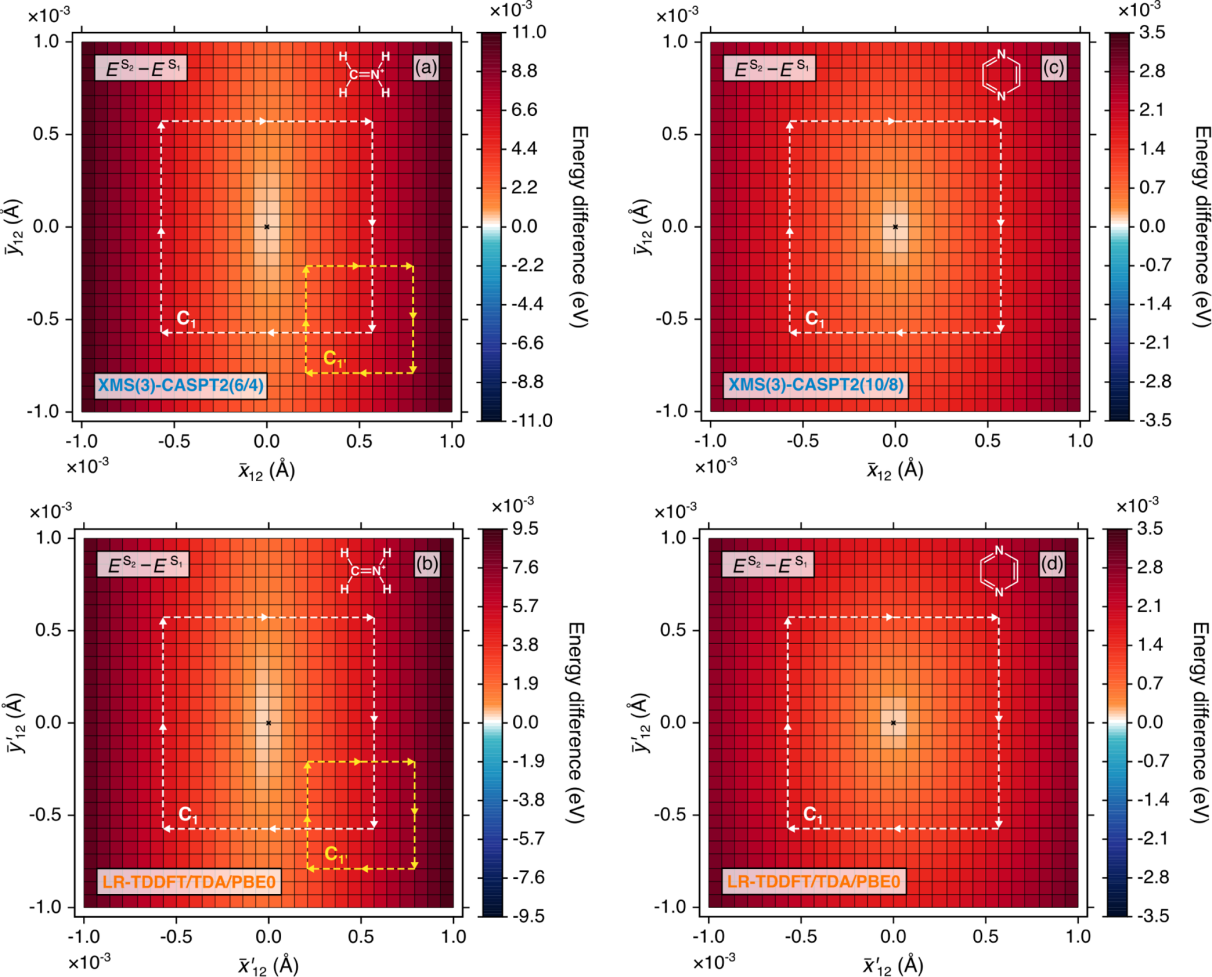
2D color map of the electronic energy difference between S_1_ and S_2_ in the vicinity of the S_2_/S_1_ MECX in (a) protonated formaldimine with XMS(3)-CASPT2(6/4)/cc-pVTZ, (b) protonated formaldimine with LR-TDDFT/TDA/PBE0/cc-pVDZ, (c) pyrazine with XMS(3)-CASPT2(10/8)/cc-pVTZ, and (d) pyrazine with LR-TDDFT/TDA/PBE0/cc-pVDZ. The dashed arrows indicate the direction of the closed paths, *C*_1_ and *C*_1′_, along which γ_*n*_ in eq 1 is evaluated, see Table 1 for numerical values. The black cross indicates the location of the optimized MECX geometry. The Lewis structures of both molecules are given as an inset in each plot.

**Figure 2:**
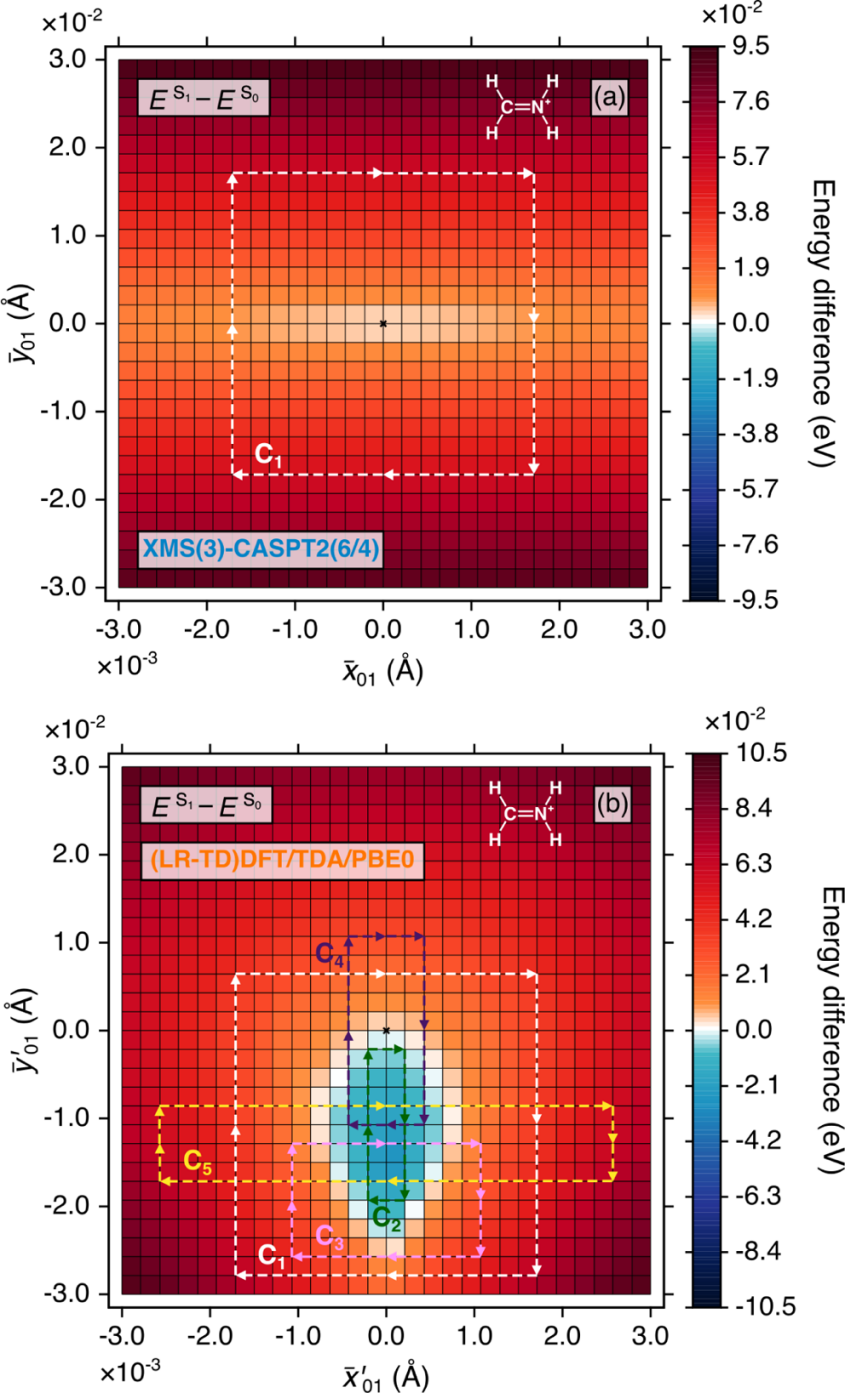
2D color map of the electronic energy difference between S_0_ and S_1_ in the vicinity of the S_1_/S_0_ MECX (or MECP) in protonated formaldimine along an extended branching plane with (a) XMS(3)-CASPT2(6/4)/cc-pVTZ and (b) (LR-TD)DFT/TDA/PBE0/cc-pVDZ. The dashed arrows indicate the direction of the closed paths, *C*_1_ to *C*_5_, along which γ_*n*_ in eq 1 is evaluated, see Table 2 for numerical values. The black cross indicates the location of the optimized MECX (or MECP) geometry. The Lewis structure of protonated formaldimine is given as an inset in each plot.

To evaluate γ_*n*_ in eq 1, we defined a closed rectangular^36,37^ path (see Figures 1 and 2), utilizing the precomputed grid of geometries that defined the respective MECX (or MECP) branching plane. The total line integral in eq 1 was split into four separate line integrals, one along each of the four straight-line segments. For each straight-line segment, the nuclear coordinate vector, **R**, was parametrized as a linear interpolation between the initial and final geometries using a scalar parameter, 0 ≤ α ≤ 1. The integral was then transformed to an integral over α, for which the integrand is the dot product of the **d**_*ij*_(**R**) vector and the vector defining the difference between the initial and final geometries along the segment. This integral was evaluated numerically using the trapezoidal rule^70^ with the relevant grid geometries. We note that errors arising from the use of a finite grid of geometries will contribute to the discrepancies between γ_*n*_ and π (or zero), in addition to those mentioned in Section 1.

To correct random sign-flipping of the **d**_*ij*_(**R**) vectors along the closed rectangular paths in protonated formaldimine, we first inspected the sign of a related quantity, namely the transition dipole moment (TDM), along the same path and manually flipped the sign of the TDM at a given geometry to ensure that it varied smoothly (i.e., it was continuous) as a function of the nuclear coordinates. At the same geometries, we then manually corrected the sign of the corresponding **d**_*ij*_(**R**) vector. The same procedure could not be used for pyrazine because the TDM is zero by symmetry. Instead, we manually corrected the sign of **d**_*ij*_(**R**) in order to ensure that the dot product between **d**_*ij*_(**R**) vectors computed at subsequent geometries along the closed rectangular path was positive.

## 3 Results and Discussion

### 3.1 S_2_/S_1_ MECX Branching Spaces

We start by considering the lowest two singlet excited states, S_1_ and S_2_, in protonated formaldimine, plotting the S_2_ – S_1_ energy difference in the vicinity of the S_2_/S_1_ MECX within the branching space (Figure 1a,b). The value of γ_*n*_ in eq 1 was calculated along two closed rectangular paths: (i) one centered on the optimized S_2_/S_1_ MECX geometry, i.e., point *x̅*_12_ = 0.0 Å, *y̅*_12_ = 0.0 Å (*C*_1_ in Figure 1) and (ii) the other displaced from the optimized S_2_/S_1_ MECX geometry, i.e., centered on grid point *x̅*_12_ = 0.0005 Å, *y̅*_12_ = −0.0005 Å (*C*_1′_ in Figure 1). In the former case, the MECX is enclosed by the path, whereas in the latter, it is not. It is therefore expected that in the first (second) case when eq 1 is evaluated exactly along a modest-sized loop, the value of γ_*n*_ should be close to π (zero). Indeed, this is what is observed for XMS-CASPT2, as is evident from the corresponding values of γ_*n*_ reported in Table 1.

**Table 1:** Values of γ_*n*_ (equation 1) along the Closed Paths *C*_*n*_ (Figure 1) on the Branching Plane of the S_2_/S_1_ MECXs in Protonated Formaldimine and Pyrazine^a^

protonated formaldimine	XMS(3)-CASPT2(6/4)	LR-TDDFT/TDA/PBE0
*C*_1_	0.99967	1.00050
*C*_1′_	0.00021	0.00172

pyrazine	XMS(3)-CASPT2(10/8)	LR-TDDFT/TDA/PBE0
*C*_1_	0.99932	0.99916

a Values are reported in units of π.

As alluded to in Section 1, the situation for LR-TDDFT/TDA/PBE0 is arguably not as clear. Between excited electronic states, **d**_*ij*_(**R**) vectors computed within linear-response TDDFT can only ever be approximate (even in the limit that the linear-response formalism is, itself, exact) due to the formal requirement of needing to go to quadratic response. Therefore, should it be expected that the LR-TDDFT/TDA/PBE0 **d**_12_(**R**) vectors match the correct behavior of those of XMS-CASPT2? Inspecting Table 1, the answer to this question is Yes; eq 1 calculated using LR-TDDFT/TDA/PBE0 quantities appropriately returns values of γ_*n*_ close to π and zero, for paths *C*_1_ and *C*_1′_, respectively. For these two states, which are admittedly dominated by single excitations, LR-TDDFT/TDA unequivocally provides not only the correct topology of CXs between excited electronic states (as discussed in ref (21).) but also shows the correct physics (i.e., the topological phase) in their vicinity. The success of LR-TDDFT/TDA/PBE0 at reproducing the topological phase upon adiabatic transport around a CX between two excited electronic states is further corroborated by considering the S_2_/S_1_ MECX in pyrazine (Figure 1c,d). Again, both XMS-CASPT2 and LR-TDDFT/TDA/PBE0 correctly predict a value of γ_*n*_ close to π for a path enclosing the MECX—see Table 1.

### 3.2 S_1_/S_0_ MECX (or MECP) Branching Spaces

We now focus our attention on the S_1_/S_0_ MECX in protonated formaldimine. For direct comparison between both methods, we plotted the XMS-CASPT2 S_1_ – S_0_ energy difference along an extended branching plane (±0.003 × **x̅**_01_(**R**) and ±0.03 × **y̅**_01_(**R**)), the same dimensions as required to observe the S_1_/S_0_ intersection ring in (LR-TD)DFT/TDA/PBE0 (Figure 2). Again for XMS-CASPT2, the value of γ_*n*_ for a rectangular path enclosing the S_1_/S_0_ MECX geometry (*C*_1_ in Figure 2a) is correctly close to π (Table 2). This is unsurprising given that XMS-CASPT2, a multireference electronic structure method, has no difficulty in describing CXs involving the ground electronic state.

**Table 2:** Values of γ_*n*_ (Equation 1) along the Closed Paths C_*n*_ (Figure 2) on the Branching Plane of the S_1_/S_0_ MECX (or MECP) in Protonated Formaldimine^a^

protonated formaldimine	XMS(3)-CASPT2(6/4)	(LR-TD)DFT/TDA/PBE0
*C*_1_	1.00205	0.00029
*C*_2_		0.00620
*C*_3_		0.00811
*C*_4_		0.00807
*C*_5_		0.00066

a Values are reported in units of π.

Predicting the influence of the S_1_/S_0_ intersection ring in (LR-TD)DFT/TDA/PBE0, however, is less trivial, considering that it comprises an infinite number of degeneracy points, as opposed to a single point of degeneracy. Williams et al.^48^ showed that the defective CXs between excited electronic states in EOM-CCSD, which show a similar ring-like intersection (although for different reasons^a^), do in fact reproduce the topological phase effect, giving hope for the application of this method in excited-state dynamics simulations, provided the nuclear wave packet never ventures too close to the defective excited-to-excited state CX. We therefore consider whether (LR-TD)DFT/TDA/PBE0 shows a similar positive behavior for the intersection ring between the ground and first excited electronic states despite its incorrect dimensionality. Evaluating γ_*n*_ for a path enclosing the entire S_1_/S_0_ intersection ring (*C*_1_ in Figure 2b) within the extended branching plane yields a value close to zero (Table 2), suggesting that (LR-TD)DFT/TDA/PBE0 fails to reproduce the topological phase.

In order to give a fair judgment of (LR-TD)DFT/TDA/PBE0, however, we ask a follow-up question. Do we obtain a value of γ_*n*_ close to π for (LR-TD)DFT/TDA/PBE0 if we instead consider a closed path fully inside the S_1_/S_0_ intersection ring (i.e., the region of negative excitation energies)? It could be argued that defining such a path would enclose only a single point of interest related to the tip of one (or both) of the interpenetrating cones (i.e., the supposed geometry of the hypothetical true CX) but would exclude the—to again use the language of Williams et al.^48^—defective ring of infinite degeneracy points. We therefore looked at a second path, *C*_2_, which is now completely within the intersection ring (Figure 2b); however, once again, we obtained a value of γ_*n*_ close to zero (Table 2).

Finally, we also considered three further cases where only a portion of the intersection ring is enclosed by the path: (i) C_3_, which crosses the intersection ring twice along one side of the rectangle; (ii) C_4_, which crosses the intersection ring once on one side and once on the opposite side of the rectangle; and (iii) C_5_, which crosses the intersection ring four times. In all cases, γ_*n*_ is close to zero (Table 2).

Our results indicate that even though (LR-TD)DFT/TDA/PBE0 exhibits something reminiscent of two interpenetrating cones (or even an “approximate” CX^15^) between the ground and first excited electronic states in protonated formaldimine, it does not recover anything resembling the topological phase for any of the closed paths considered.

## 4 Conclusions

This work shows that AA LR-TDDFT/TDA/PBE0 is able to accurately reproduce the topological phase accumulated by the adiabatic electronic wave function along a path enclosing the S_2_/S_1_ MECX in protonated formaldimine and pyrazine despite the use of approximate linear-response (rather than the appropriate quadratic-response) TDDFT **d**_*ij*_(**R**) vectors. The observation provides further evidence that AA LR-TDDFT/TDA offers a reasonable description of CXs between excited electronic states not only with respect to CX topology and topography, as previously illustrated in ref (21), but also with respect to the physics within the immediate vicinity of the degeneracy point. This provides further confidence in the use of AA LR-TDDFT/TDA for excited-state dynamics simulations involving states of predominantly single-excitation character.

For a path enclosing the S_1_/S_0_ intersection ring exhibited by AA (LR-TD)DFT/TDA/PBE0 in protonated formaldimine, our findings are less fruitful: the topological phase is not reproduced. The same observation is made for a path fully inside the intersection ring and for three paths that cross it. Nonetheless, further investigation is still needed to see whether the lack of the correct topological phase behavior by the S_1_/S_0_ intersection ring in AA (LR-TD)DFT/TDA/PBE0 drastically affects the accuracy of nonadiabatic dynamics simulations of protonated formaldimine, especially considering the number of different strategies employed in surface-hopping dynamics simulations to hop between electronic states in the vicinity of CXs. This is the topic of ongoing work.

## Supporting Information Available

All optimized MECX (or MECP) geometries and corresponding branching space vectors presented in this work, together with the geometries considered along each of the closed paths (ZIP)

## Footnotes

a We note that while it is possible for excitation energies in full LR-TDDFT to erroneously become imaginary at certain nuclear geometries, invoking the TDA (as was done in the present study) forces such excitation energies to be strictly real but still possibly negative.^23,71,72^ As such, differences in the behavior of the (LR-TD)DFT/TDA ground-to-excited state defective CX here and the EOM-CCSD excited-to-excited state defective CX in ref (48). are perhaps not that surprising.
